# Utilizing Information Bottleneck to Evaluate the Capability of Deep Neural Networks for Image Classification †

**DOI:** 10.3390/e21050456

**Published:** 2019-05-01

**Authors:** Hao Cheng, Dongze Lian, Shenghua Gao, Yanlin Geng

**Affiliations:** 1 Shanghai Institute of Microsystem and Information Technology, Chinese Academy of Sciences, Shanghai 200050, China; 2 University of Chinese Academy of Sciences, Beijing 100049, China; 3 School of Information Science and Technology, ShanghaiTech University, Shanghai 201210, China; 4 State Key Laboratory of ISN, Xidian University, Xi’an 710071, China

**Keywords:** information bottleneck, mutual information, neural networks, image classification

## Abstract

Inspired by the pioneering work of the information bottleneck (IB) principle for Deep Neural Networks’ (DNNs) analysis, we thoroughly study the relationship among the model accuracy, I(X;T) and I(T;Y), where I(X;T) and I(T;Y) are the mutual information of DNN’s output *T* with input *X* and label *Y*. Then, we design an information plane-based framework to evaluate the capability of DNNs (including CNNs) for image classification. Instead of each hidden layer’s output, our framework focuses on the model output *T*. We successfully apply our framework to many application scenarios arising in deep learning and image classification problems, such as image classification with unbalanced data distribution, model selection, and transfer learning. The experimental results verify the effectiveness of the information plane-based framework: Our framework may facilitate a quick model selection and determine the number of samples needed for each class in the unbalanced classification problem. Furthermore, the framework explains the efficiency of transfer learning in the deep learning area.

## 1. Introduction

### 1.1. Deep Neural Networks

Deep neural networks (DNNs) are very powerful machine learning models that have revolutionized many research and application areas [1,2,3,4,5,6] in recent years. These include image recognition [1,4], speech recognition [2], and natural language processing [5]. DNN is a type of representation learning that can automatically generate good representations from raw data for further processing. With many training data and GPU acceleration [1], the performance of DNN has greatly exceeded other traditional learning algorithms in image recognition tasks. Inspired by the work in [1], researchers have developed more efficient network structures: The work in [7] decreased the size of kernel and found better representations of data. The work in [8] proposed a facial recognition tool, named FaceNet, that could be embedded for face recognition and clustering. The work in [9] proposed ResNet by utilizing identity mapping between layers. ResNet could make the network deeper than 1000 layers without losing performance and has been widely used in many applications.

However, DNN has millions of parameters and numerous hyper-parameters. Before training a DNN model, one needs to define all the hyper-parameters heuristically. These include the initial learning rate, momentum, batch size, activation functions, number of layers, weight decay, etc. There is no standard guideline on how to choose appropriate hyper-parameters. Furthermore, the same network structure or hyper-parameter setting may be suitable for one task (dataset), but fail on the other one. Thus, for DNN, it lacks deep understanding or an explanation of how networks work or behave.

Recently, there have been some works that tried to understand networks by visualization. The work in [10] proposed a method to compute the image-resolution receptive field of neural activations in a feature map. Accurate calculation of the receptive field would help people to understand the representation of the filter. The work in [11,12] understood DNN by estimating the feature distribution of each class in the feature space of the pre-trained model. The work in [13] used the decovnet operation for visualizing discriminant features from data. The work in [14] introduced a tool, named network dissection. This tool can be used to quantify the exploitability of hidden representations of kernels in convolutional networks by evaluating the alignment between individual hidden units and a set of semantic concepts. However, almost all of these visualization techniques understand DNNs by visualizing features or activation maps to gain insights into neural networks, which somehow lacks a theoretical basis. In this work, we are going to use the tool of mutual information, which is a fundamental measure in information theory, to represent networks in the information plane for better understanding.

**Remark** **1.** *This is an extend version of our conference paper published in ECCV 2018 [15].*

### 1.2. Mutual Information and Information Bottleneck

The reason we used mutual information is that it provides a measure to quantify the common part between two random variables, especially for the case where their samples are highly non-linear. Notice that supervised or unsupervised deep learning models have the input data and the output ones; the relationship or uncertainty between them can be measured by mutual information. For example, the work in [16] introduced a new learning rate schedule based on mutual information. The work in [17] utilized a mutual information method to score all the candidate features in the network and selected the most informative one as the input for DNN. The work in [18] learned a better representation by maximizing the mutual information between DNN’s input and output.

In supervised deep learning, the label *Y* is also available. In addition to the relationship between the input *X* and output *T*, the correlation between the output *T* and label *Y* is also worth studying. We used I(X;T) and I(T;Y) to represent the corresponding mutual information. The study of I(X;T) and I(T;Y) inspires the information bottleneck (IB) method. IB was introduced by Naftali Tishby et al. [19] and serves as a method for compressing the source data (random variable) without losing the predictive capability for the target label (random variable). Since then, the modification and generalization of IB has attracted considerable interest. The work in [20] generalized the IB method to the continuous setting, which deals with variables such as Gaussian. The work in [21] proposed the deterministic IB (DIB), a different formulation that uses entropy instead of mutual information. It stated that DIB could better distinguish relevant features. The work in [22] deeply explored the relationship between IB and minimal sufficient statistics.

In recent years, IB has been applied to the deep learning research field. The work in [23] introduced a variational approximation technique to IB. The new method uses a neural network to parameterize the IB model, which could lead to an efficient and stable training process. The work in [24] introduced a new IB-based framework, called parametric IB (PIB). The advantage of PIB is that it could jointly optimize the compressed information and the relevant information of the layers in the neural network and leads to a better representation ability of DNNs. The work in [25] proposed a method called information dropout, which generalizes the original dropout with theoretical consideration. The new dropout method can make it easier for the network to fit the training data.

### 1.3. Visualizing DNN via Information Bottleneck

**Figure 1:**
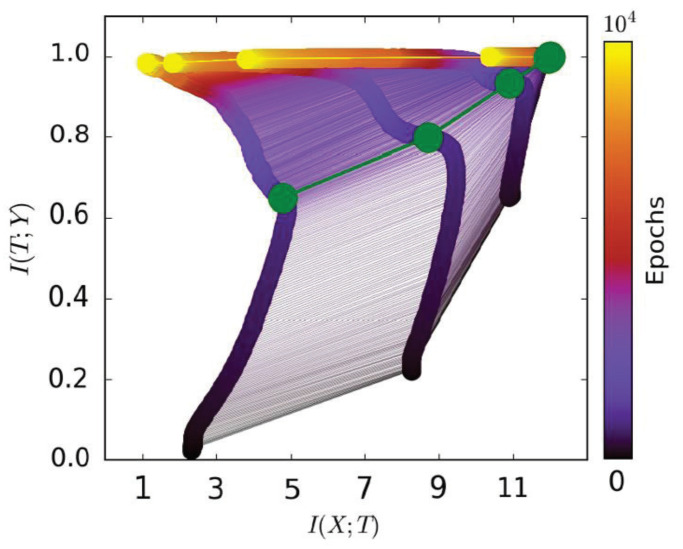
In this figure (from [15]), the network that performs the experiment is a fully-connected neural network. The input data *X* are a 12-dimensional binary vector. The label *Y* takes values from {0, 1}. Each mutual information path corresponds to a hidden layer. The leftmost path represents the last hidden layer, and the rightmost path represents the first hidden layer. The green points (also called transition points) separate mutual information paths into two stages. Best viewed in color.

Different from the work in [23,24,25], Ravid and Naftali [26] took IB as a visualization tool to understand how neural networks work in general. Specifically, they estimated the mutual information I(X;T) and I(T;Y) for **each hidden layer**, where *X* is the input data, *Y* is the label, and *T* is the hidden layer output, respectively. Figure 1, from [15], draws I(X;T), I(T;Y) with training epochs in the two-dimensional plane (referred to as the information plane). From the figure, the green points (transition points) separate mutual information paths into two stages:

- The first phase, which is also called the “fitting phase”, only takes a little time in the whole training procedure. I(X;T) and I(T;Y) both increase in this stage. It can be explained as: the network needs to remember the information from the data “I(X;T)” for fitting the label “I(T;Y)”.
- The second phase, which is also called the “compression phase”, takes most of the training time. I(T;Y) still increases while I(X;T) decreases. It can be explained as: the value of I(X;T), obtained in the first fitting phase, contains too much irrelevant information (for classification). The network needs to drop this irrelevant information for better generalization.

The dynamic change of I(X;T) and I(T;Y) in the information plane helps us understand how neural networks work. However, there are still some unsolved problems: (1) “Accuracy” is an important indicator for DNNs. To evaluate DNNs via IB, one needs to investigate fully the relationship among the model accuracy, I(X;T), and I(T;Y). One benefit of this investigation is that we can use the information plane to help us evaluate or select a better model that leads to a higher accuracy in a short time (see Section 3.3). (2) The models used in [26] are some simple fully-connected neural networks (FCs), while convolutional neural networks (CNNs) are generally used in many applications, like computer vision and speech recognition. Is there any difference between FCs and CNNs in the information plane? (3) Could IB be used in some other problems arising in deep learning? This paper aims to address these problems to make IB theory more acceptable and applicable in deep learning research. The contributions of our work are summarized in two parts as follows:

(1) Framework:
  - “Accuracy” is a common indicator that reflects DNN’s quality. Since we are to use mutual information to evaluate DNNs, we need to explore the relationship among I(X;T), I(T;Y), and accuracy. With extensive experiments, we found that low I(X;T) and high I(T;Y) both contribute to the accuracy. Furthermore, the correlation between I(X;T) and accuracy grew stronger as the network got deeper.
  - An information plane-based framework is proposed based on the investigation of the relationship among I(X;T), I(T;Y), and accuracy. We used the framework to evaluate fully-connected neural networks (FCs) and convolutional neural networks (CNNs) in the information plane and found some interesting phenomena. For example, when a dataset is harder to recognize, FCs with fewer layers do not exhibit the second phase.
  - Our results suggest that the information plane is more informative than the loss curve; thus, it may be used for better evaluation of neural networks. For example, two models with similar loss curves (both decreasing along with the training epochs) behave differently in the information plane.
(2) Applications:
  - We successfully applied the framework to the image classification problem with an unbalanced data distribution. The framework helped us determine the samples of each class needed in the image classification task.
  - We applied the framework to explain the efficiency of transfer learning. We found that the weight-transfer method helped the model fit the data only in the first learning phase. This result observed by us is consistent with [27]. While different, we verify this result from the information plane.
  - We apply the framework to analyze how different optimization methods affect DNNs. We compare the optimization methods “SGD” and “Adam” in the information plane. We found that Adam might have the advantage of fitting labels in the beginning of training, but generalizes more poorly than SGD in the end. This finding is also consistent with [28].

Compared to our original conference version [15], this paper focuses more on application scenarios arising in deep learning and image classification problems like transfer learning and on the comparison of different optimization algorithms listed above.

## 2. Materials and Methods

In this section, we introduce the definition of mutual information and explain representation learning in the language of information bottleneck. Then, we show how to estimate mutual information in DNNs in detail.

### 2.1. Some Concepts of Mutual Information

The mutual information of two discrete random variables *X* and *Y* is defined as:

(1) $$I\left(X;Y\right)=\sum_{x,y}p\left(x,y\right)\log\frac{p(x,y)}{p(x)p(y)},$$

where p(x,y) is the joint probability mass function of *X* and *Y* and p(x) and p(y) are the marginal probability mass functions of *X* and *Y*, respectively.

For continuous random variables, the summation is replaced by a definite double integral:

(2) $$I\left(X;Y\right)=\int_{y}\int_{x}p\left(x,y\right)\log\frac{p(x,y)}{p(x)p(y)}dxdy,$$

where p(x,y), an abuse of notation, is now the joint probability density function of *X* and *Y*. Compared with (2), (1) is easier and more practical to calculate (integrating probability density functions is time consuming in networks). Thus, in the experiments, we will first on using a discretization technique to transform continuous values to discrete ones before calculating the mutual information. The detail can be found in Section 2.3.

For a discrete *X*, the entropy H(X) can be written in the form of mutual information:

(3) $$H\left(X\right)=I\left(X;X\right)=-\sum_{x}p\left(x\right)\log p\left(x\right).$$

In general, mutual information quantifies the similarity, or dependence, between two variables. The following two properties of mutual information are essential for analyzing neural networks.

- Function transformation: For any invertible functions ψ and ϕ,
  (4) I(X;Y)=I(ψ(X);ϕ(Y)).
- Data processing inequality: If X→Y→Z forms a Markov chain, then:
  (5) I(X;Y)≥I(X;Z).

### 2.2. Optimal Representations of Information Bottleneck

In machine learning, representation learning is a set of techniques that allows a system to discover the representations needed for feature detection or classification from raw data automatically. The neural network is a type of representation learning that can be used to perform feature learning, since it learns a representation of the input at the hidden layer, which is subsequently used for classification or regression at the output layer. The goal of representation learning is to extract the efficient representation of raw data *X*. This is often called “compression”. However, the compression should not lose the predictive capability of the label *Y*. This learning process is identical to the concept of minimal sufficient statistics. A minimal sufficient statistic T(X) is the solution to the following optimization problem:

(6) $$T\left(X\right)=\underset{S(X):I(S(X);Y)=I(X;Y)}{\arg\min}I\left(S\left(X\right);X\right).$$

From (6), the minimum T(X) is the simplest sufficient statistic. In other words, T(X) is the function of other sufficient statistics. One way of formulating this is through the Markov chain Y→X→S(X)→T(X). Thus, from the concept of minimal sufficient statistics, the goal of DNNs is to make I(X;S(X)) as small as possible, which means the representation is efficient; while I(S(X);Y) should be the same value of I(X;Y), which means the information on *Y* is not reduced. Since explicit formulae for minimal sufficient statistics only exist for very special distributions (exponential families), the work in [19] relaxed this optimization problem by first allowing the map to be stochastic defined as an encoder P(T|X) and, then, by allowing the map to capture I(X;T) as much as possible, not necessarily all of it. This leads to the IB method [19].

IB is a special case of the rate-distortion theory, which provides a technique for finding approximate minimal sufficient statistics, or the optimal trade-off between the compression of *X* and the predictive ability of *Y*. Let *x* be an input, *t* be the output of the model, or the compressed representation of *x*, and p(t|x) be a probabilistic mapping. We can formulate the information bottleneck as the following optimization problem:

(7) $$\min_{p(t|x),Y\to X\to T}\left\{I\left(X;T\right)-\beta I\left(T;Y\right)\right\}.$$

The positive Lagrange multiplier β operates as a trade-off parameter between the compression I(X;T) and the amount of preserved relevant information I(T;Y). The solution to (7) can be given by three IB self-consistent equations:

(8) $$\begin{array}{ccc} p(t|x) & = & \frac{p(t)}{Z(x;\beta )}\exp\{-\beta D_{KL}(p\left(y|x\right)||p\left(y|t\right))\}, \end{array}$$

(9) $$\begin{array}{ccc} p(t) & = & \sum_{x}p\left(t|x\right)p\left(x\right), \end{array}$$

(10) $$\begin{array}{ccc} p(y|t) & = & \sum_{x}p\left(y|x\right)p\left(x|t\right), \end{array}$$

where Z(x;β) is the normalization function.

From (7), a good representation is a trade-off between I(X;T) and I(T;Y). These two terms are essential for analyzing neural networks. In the next section, we will show how to estimate mutual information in neural networks approximately.

### 2.3. Calculating Mutual Information in DNNs

In Section 2.2, we emphasize that I(X;T) and I(T;Y) are important for analyzing DNNs (a type of representation learning model). Next, we will show step by step how to calculate mutual information I(X;T) and I(T;Y) in neural networks.

**Figure 2:**
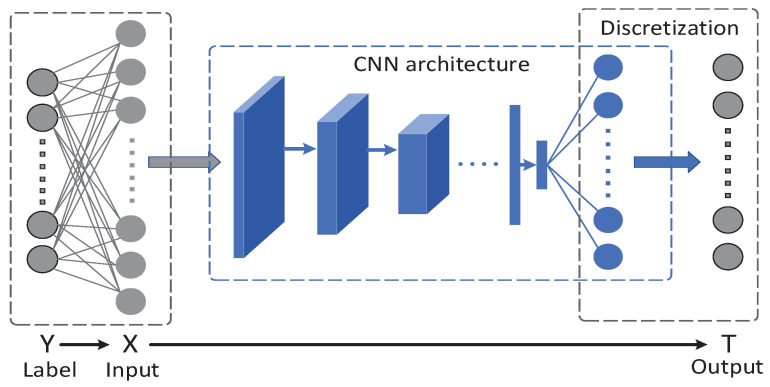
This figure, from [15], depicts a general neural network structure. The blue circles represent the softmax probability. Discretization is performed on the output of this softmax function.

First, Figure 2 shows a general network structure. Y→X→T forms a Markov chain. The number of neurons in *T* equals the number of classes *C* in the training set. Notice that the outputs of *T* are the scores of different classes and are unbounded. To better calculate mutual information I(X;T) and I(T;Y), a normalized exponential function is employed to squash a *C*-dimensional real vector *z* of arbitrary real values to a *C*-dimensional vector σ(z) of real values in the range [0,1] that add up to one. The normalization function is written as:

(11) $$\sigma {\left(z\right)}_{j}=\frac{e^{z_{j}}}{\sum_{i=1}^{C}e^{z_{i}}}\;\mathrm{for}\;j=1,\cdots ,C,$$

which is of the same form as the softmax function in the neural network. Now, in the layer *T*, the value of each neuron ranges from 0–1. Then, we bin each neuron’s output into 10 equal intervals between zero and one and get our final model output *T*, which is the discretization process shown in Figure 2.

Second, from (1), one needs to calculate the joint distribution of two random variables for estimating mutual information. Suppose we want to estimate I(X;T) from a mini-batch (containing *n* samples) in the image classification task; since every image is different from other images, for the *i*th input image $x_{i}$, it is common to let $p\left(x_{i}\right)=\frac{1}{n}$. When performing calculation of mutual information at each epoch, the network’s parameter θ is fixed. $t_{i}$ is generated by inputting $x_{i}$ into the deterministic network. We can put is as $t_{i}=f\left(x_{i},\theta \right)$. In this case, $p(t_{i}|x_{i})=1$. There are multiple data points; thus, one can take *X* as a random variable (of data), but not for θ at this epoch since it is fixed. Thus, *T* is a deterministic function of *X*. Therefore, we have H(T|X)=0. At the next epoch, the network’s parameter θ is updated, and we continue this calculation process. Now, due to binning, we have:

(12) $$\begin{array}{c} I\left(X;T\right)=H\left(T\right)-H\left(T|X\right)=H\left(T\right)=\sum_{t}p\left(t\right)\log\frac{1}{p(t)}=\sum_{i=1}^{n}\frac{1}{n}\log\frac{1}{p(t_{i})}. \end{array}$$

From (12), I(X;T) is related to the distribution of *T*. It does not mean that calculating I(X;T) does not need *X*, since *T* is generated by inputting *X* into the network. Suppose in the mini-batch, after binning, each value $t_{i}$ is different from others. Then, I(X;T) achieves the maximum logn. Suppose some $t_{i}$’s have the same value, then I(X;T) becomes lower. Furthermore, (12) shows that I(X;T) is not related to the dimension of images. Thus, for high dimensional images, we can still estimate mutual information this way (usually, the number of classes is much smaller than the dimension of the image). However, there are several points worth noticing:

- We make a hypothesis that in the mini-batch, each image is different from the others. Actually, it can happen that some images are the same. However, this will not affect the expression (12), since the same images must produce the same outputs. Only the probability mass function of *T* might be changed.
- The mutual information we estimate may be different from the ground truth since we perform discretization on the layer *T*. Thus, for fair comparison, for different data with the same number of classes, we need to use the same discretization interval.
- If the number of neurons in the layer *T* is large, the mutual information I(X;T) barely changes; since in this case, the sample space of *T* is huge even if we decrease the number of intervals. As a result, X→T is approximately a bijection function (p(x|t) is almost deterministic). Thus, I(X;T)≈H(X) from (1) and (3).

From the above analysis, the dimension of *T* cannot be huge. Since the number of neurons in the layer *T* equals the number of classes in the training set, our method is subject to the number of classes. For cases with a large numbers of classes, we investigated the method of clustering to estimate the probability mass functions. We provide an API at https://github.com/piratehao/API-of-estimating-mutual-information-in-networks.

## 3. Main Results

This section is structured as follows: In Section 3.1, we explore the relationship among the model accuracy, I(X;T), and I(T;Y); in Section 3.2, we introduce our information plane-based framework and utilize this framework to compare the differences between FCs and CNNs in the information plane; in Section 3.3, we compare the evaluation framework with loss curves and state that our framework is more informative when evaluating neural networks; in Section 3.4, we evaluate modern popular CNNs in the information plane; in Section 3.5, we show that the information plane can be used to evaluate DNNs, not only on a dataset, but also on each single class of the dataset in image classification tasks; in Section 3.6, we use IB to evaluate DNNs for image classification problems with an unbalanced data distribution; in Section 3.7, we analyze the efficiency of transfer learning in the information plane; in Section 3.8, we compare different optimization algorithms of neural networks in the information plane.

### 3.1. Relationship among Model Accuracy, I(X;T), and I(T;Y)

Accuracy is a common “indicator” to reflect the model’s quality. Since we are to use mutual information to evaluate neural networks, it is necessary to investigate the relationship among I(X;T), I(T;Y), and the accuracy. The work in [22] studied the relationship between mutual information and accuracy and stated that I(T;Y) explains the training accuracy, I(X;T) serves as a regularization term that controls the generalization; while from our experiments, we found that in deep neural networks, when I(T;Y) is fixed, there is also a correlation between I(X;T) and the training accuracy, where *X* represents the training data. Furthermore, the correlation grew stronger as the network got deeper.

**Figure 3:**
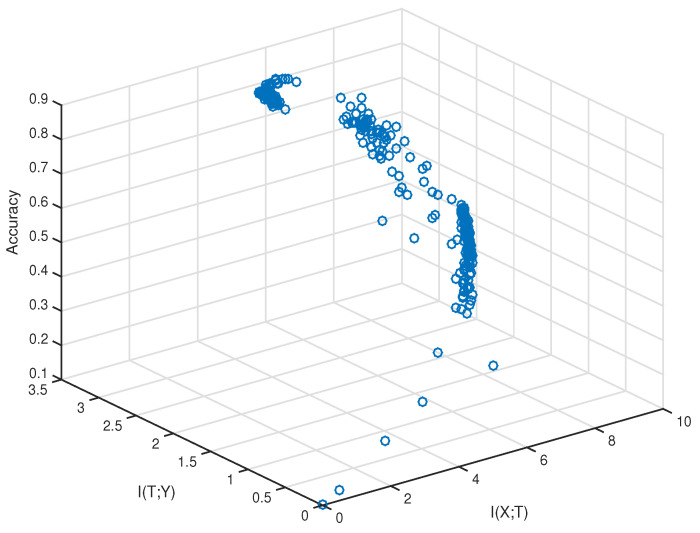
Visualization of the sampled data in 3D space.

To validate the hypothesis experimentally, we need to sample the values of the training accuracy, I(X;T), and I(T;Y). The method of estimating mutual information has been discussed in Section 2.3. The network and dataset we chose were VGG-16 and CIFAR-10, respectively. The sampling was performed after every fixed iteration steps during the training. We first plotted I(X;T), I(T;Y), and the training accuracy of sampled data in 3D space, which is shown in Figure 3.

From Figure 3, we can see the data did not fully fill the whole space. They came from a narrow area out of the whole 3D space. Furthermore, there existed two points that had similar I(T;Y), but the point with smaller I(X;T) had a higher training accuracy. To verify the correlation between (X;T) and the training accuracy quantitatively, we used the method of “checking inversion”.

First, we used $I{\left(X;T\right)}_{i}$, $I{\left(T;Y\right)}_{i}$, and $Acc_{i}$ to denote the values of the *i*th sampling result, which corresponds to a blue circle in Figure 3. Suppose there exists a sample pair (i,j) such that $I{\left(T;Y\right)}_{i}=I{\left(T;Y\right)}_{j}$, then we can directly check the relationship between I(X;T) and the training accuracy to find how they are related. However, each $I{\left(T;Y\right)}_{i}$ is a real number, and it is difficult to find samples that have the same value of I(T;Y). Thus, we verify the hypothesis by checking inversions. An inversion is defined as a pair of samples (i,j) that satisfies $I{\left(T;Y\right)}_{i}<I{\left(T;Y\right)}_{j}$ and $Acc_{i}>Acc_{j}$. A “satisfied inversion” is defined as an inversion for which $I{\left(X;T\right)}_{i}<I{\left(X;T\right)}_{j}$ and $Acc_{i}>Acc_{j}$. The “percentage”, defined as follows:

(13) $$\mathrm{percentage}=\frac{\#\mathrm{satisfied}\;\mathrm{inversions}}{\#\mathrm{all}\;\mathrm{inversions}},$$

is a proper indicator to reflect the rightness of our hypothesis that low I(X;T) also contributes to the training accuracy. Consider two cases:

- The percentage is near 0.5. This means that almost half of the inversion pairs satisfy $I{\left(X;T\right)}_{i}<I{\left(X;T\right)}_{j}$ and $Acc_{i}>Acc_{j}$, and the other inversion pairs satisfy $I{\left(X;T\right)}_{i}<I{\left(X;T\right)}_{j}$ and $Acc_{i}<Acc_{j}$. Thus, I(X;T) almost has no relation to the training accuracy.
- The percentage is high. This means that a large number of inversion pairs satisfy $I{\left(X;T\right)}_{i}<I{\left(X;T\right)}_{j}$ and $Acc_{i}>Acc_{j}$. Thus, low I(X;T) also contributes to the training accuracy.

We performed experiments under different training conditions to train the neural networks. Table 1, from [15], records percentages with different training conditions.

Table 1 shows that in various condition settings, the percentages were all over 0.5, which indicates that I(X;T) also contributed to the training accuracy. Furthermore, different training schemes (SGD, batch gradient descent (BGD)) and network structures (FC, CNN) may lead to different percentages. We explain these phenomenons as follows:

- Even though I(T;Y) represents the correlation between the model output and label, I(T;Y) is not a monotonic function of the training accuracy. Suppose there are *C* classes in the image dataset, and let $C_{i}$ be the *i*th class. Consider two cases: In the first case, T=σ(Y) where σ is an identity mapping, which indicates that the model output *T* always equals the true class. For another case, T=φ(Y) where φ is a shift mapping, which indicates that if the true class is $C_{i}$, the prediction of *T* is $C_{i+1}$. In both cases, since σ and φ are invertible functions, from (4), we have I(T;Y)=I(σ(Y);Y)=I(φ(Y);Y)=H(Y). However, in the first case, the training accuracy was one, whereas in the other case it was zero.
- The activation function of the linear network was identity mapping. Thus, the loss function of the linear network was a convex function; whereas the loss function of convolutional neural networks is highly non-convex. By using BGD to optimize a convex function with a proper learning rate, the training loss with respect to all the training data always decreased (the model was stablest in this case), which indicated that *T* was gradually closer to *Y* during training. Thus, I(T;Y) can fully explain the training accuracy and I(X;T) may not have contributed to the training accuracy greatly; however, when the loss function was a non-convex function, or the training scheme was SGD. The loss with respect to all the training data did not decrease all the time (the network was sometimes learning in the wrong direction). Thus, I(T;Y) cannot fully explain the training accuracy for SGD and convolutional neural networks.
- The work in [29] used I(X;T) to represent the stability of the learning algorithm. The model with low I(X;T) tended to be more stable on the training data. Thus, when $I{\left(T;Y\right)}_{i}$ and $I{\left(T;Y\right)}_{j}$ are equal, the model with low I(X;T) may lead to a high training accuracy.

Another interesting phenomenon is that we found that there was a correlation between percentage and the number of layers of convolutional neural networks. Table 2, from [15], shows that percentage rose as we increased the number of layers of convolutional neural networks. This result may relate to some inherent properties of CNNs, which is worth investigating for future work.

Compared to the training accuracy, we were more interested in validation accuracy. Thus, we also validated our hypothesis on the validation data where *X* represents the validation input. Table 3, from [15], shows that low I(X;T) also contributed to the validation accuracy. This observation will form the basis of our evaluation framework proposed in the next section.

### 3.2. Evaluating DNNs in the Information Plane

In Section 2.2, we explain that I(X;T) and I(T;Y) are essential for analyzing neural networks. The neural network training process can be regarded as finding a trade-off between I(X;T) and I(T;Y). Furthermore, in Section 3.1, the experiments showed that not only high I(T;Y), but also low I(X;T) contributed to the validation accuracy, where *X* represents validation input. Since I(X;T) and I(T;Y) are both indicators to reflect the model’s accuracy, we used $\frac{\Delta I(T;Y)}{\Delta I(X;T)}$ to represent the model’s learning capability in the information plane. Notice that $\frac{\Delta I(T;Y)}{\Delta I(X;T)}$ is exactly the slope of the mutual information curve. Thus, it represents the model’s learning capability at each moment.

**Figure 4:**
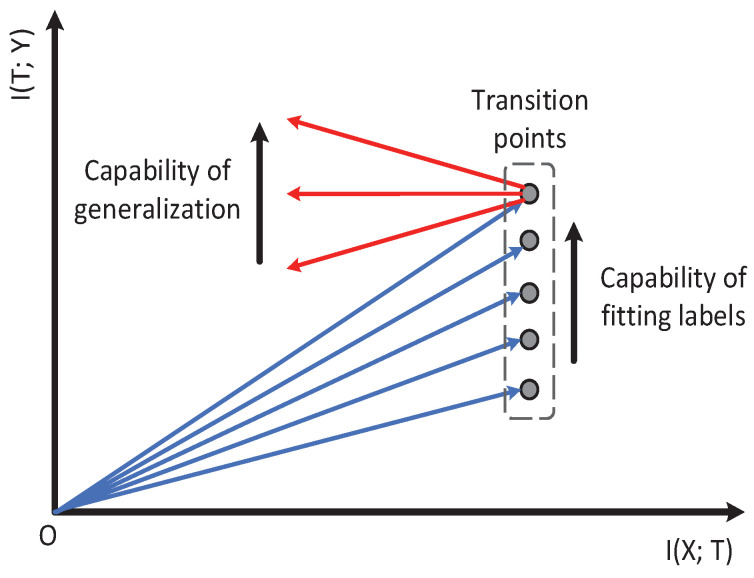
This figure (from [15]) shows the information plane-based framework. I(T;Y) before the transition point represents the model’s capability of fitting the label. $\frac{\Delta I(T;Y)}{\Delta I(X;T)}$ after the transition point represents the model’s capability of generalization.

Figure 1, from [15], shows that a mutual information path contains two learning stages. The first fitting phase only took very little time compared to the whole learning process. The model began to generalize at the second compression phase. Thus, we only used $\frac{\Delta I(T;Y)}{\Delta I(X;T)}$ to represent the model’s learning capability in the second phase. A better model is expected to have smaller (negative) $\frac{\Delta I(T;Y)}{\Delta I(X;T)}$ in the second phase; while for the first fitting phase, I(T;Y) of the model increased (the model needed to remember the training data for fitting the label). Thus, we only used I(T;Y) to represent the model’s capability of fitting the label. Based on our analysis above, we propose our information plane-based framework in Figure 4.

**Figure 5:**
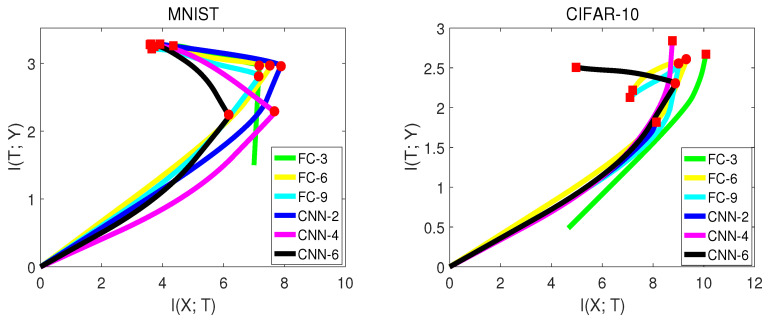
In these two figures (from [15]), FC-*i* is a fully-connected neural network with *i* hidden layers. CNN-*i* is a convolutional neural network with *i* convolutional layers.

To further explore how different datasets or network structures influence the mutual information curves in the information plane, we performed an experiment on two datasets: MNISTand CIFAR-10. The network structure contained fully-connected neural networks and convolutional neural networks with various numbers of layers. Notice for subsequent experiments, *X* in I(X;T) represents the validation input, since we cared about the validation accuracy rather than training accuracy. Furthermore, we smoothed the mutual information curves in the information plane for simplicity and better visualization. Table 4 and Figure 5, from [15] show the experiment results.

There are some interesting observations from Figure 5:

- Not all networks had exactly the second compression phase. For the MNIST experiment, all networks had two learning stages. However, for CIFAR-10, the networks with fewer hidden layers (FC-3, CNN-2, CNN-4) did not show the second phase. The reason is that CIFAR-10 is a more difficult dataset for the network to classify. The network with fewer hidden layers may not have enough generalization capability.
- Convolutional neural networks had better generalization capability than fully-connected neural networks by observing $\frac{\Delta I(T;Y)}{\Delta I(X;T)}$ in the second phase. This led to higher validation accuracy. However, I(T;Y) of convolutional neural networks was sometimes lower than fully-connected neural networks by comparing I(T;Y) at the transition point, which indicates that fully-connected networks may have a better capability of fitting the labels than convolutional neural networks in the first phase. The fact that fully-connected neural networks have a large number of weights may contribute to this.
- Not all networks have increasing I(T;Y) in the second phase. For CIFAR-10, I(T;Y) of FC-6 and FC-9 dropped down in the second phase. One possible reason is that FC with more layers may over-fit the training data.

### 3.3. Informativeness and Guidance of the Information Plane

There does not exist a network that is optimal for all problems (datasets). Usually, researchers have to test many neural network structures on a specific dataset. The network is chosen by comparing the final validation accuracy of each network. This process is time consuming since we have to train each network until convergence. In this section, we will show that the information plane could ease this searching process and facilitate a quick model selection of neural networks.

**Figure 6:**
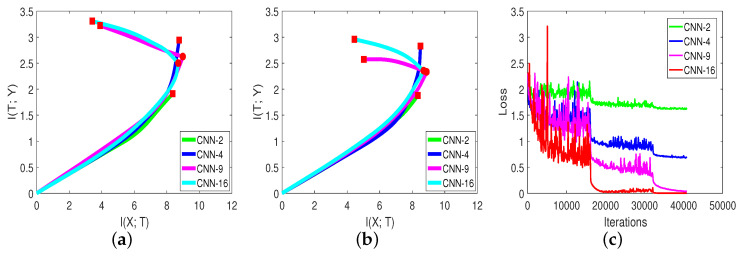
In these three figures (from [15]): (**a**) Mutual information paths of neural networks on the training set of CIFAR-10. (**b**) Mutual information paths of neural networks on the validation set. (**c**) Training losses of neural networks with training iterations.

In Section 3.2, we show that $\frac{\Delta I(T;Y)}{\Delta I(X;T)}$ can represent the capability of generalization at each moment in the second compression phase. Thus, a direct way to select the model is to compare each model’s $\frac{\Delta I(T;Y)}{\Delta I(X;T)}$ at the beginning of the compression phase. Since the first compression phase only takes a little time, we can select a better model in a short time. Figure 6 and Table 5, from [15], show the mutual information paths and validation accuracies of different networks on the CIFAR-10 dataset.

We can gain some information from Figure 6 and Table 5:

- $\frac{\Delta I(T;Y)}{\Delta I(X;T)}$ can be used to select a better network. We can compare CNN-9 and CNN-16 in Figure 6b. The slope of the mutual information curve of CNN-16 was smaller (negative) than CNN-9, which represents a better generalization capability. The final validation accuracy in Table 5 is consistent with our analysis. Thus, for a specific problem, we can visualize each model’s mutual information curve on the validation data to select a better model quickly.
- The information plane is more informative than the loss curve. By comparing Figure 6c with Figure 6a,b, we can see that the training loss of each model continued to decrease with training steps. However, the mutual information curves behaved differently. The model with fewer layers did not clearly show the second compression phase. Thus, the information plane could reveal more information about the network.
- I(X;T) mostly contributed to the accuracy in the second phase. We record the percentage of each network in Table 5. From Table 5, when the network had fewer layers, the mutual information path did not clearly show the second phase, and the percentage was low; whereas for the network that had the compression phase, the percentage was high. One possible reason is that in the second phase, the model learned to generalize (extract common features from each mini-batch). The value of percentages indicates that the compression happened even when I(T;Y)’s remained the same.

We can view I(X;T) and I(T;Y) as: I(T;Y) determines how much the knowledge *T* has about the label *Y*, and I(X;T) determines how easy this knowledge can be learned by the network.

### 3.4. Evaluating Popular CNNs in the Information Plane

**Figure 7:**
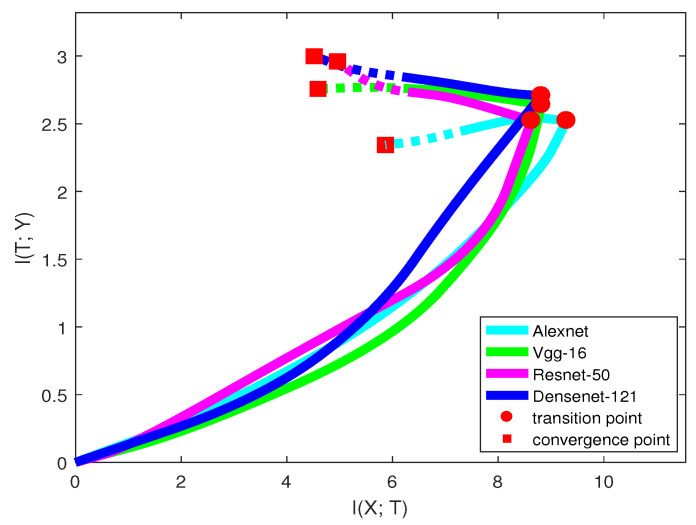
Mutual information paths of different network architectures on the CIFAR-10 dataset. For each mutual information path, training time spent on the dotted line was three-times longer than the time spent on the solid line.

The architecture of neural networks has undergone substantial development. From AlexNet [1], VGG [7] to ResNet [9] and DenseNet [30], researchers have made great efforts in searching for efficient networks. In this section, we visualize these popular networks in the information plane. Figure 7 and Table 6 show the information curve, training epochs, and accuracy of each network structure on the CIFAR-10 dataset.

Only looking at the solid line in Figure 7, we may infer that when classifying the CIFAR-10 dataset, AlexNet was the worst neural network among all the models. AlexNet has a low capability of fitting the labels, and after the transition point, I(T;Y) of the model even dropped down, indicating losing information on labels. VGG had a stronger capability of fitting labels than ResNet, but the generalization capability was relatively lower. From the trend of the curves, I(T;Y) of ResNet may be larger than VGG in the future. DenseNet was better than all the other models in both two learning phases. The model accuracies in Table 6 are consistent with our analysis.

Here, we emphasize that our prediction may not always be true, since the mutual information path may have a larger slope change in the future. See ResNet in Figure 7. Thus, there existed a trade-off between the training time and confidence of our prediction. We can make a more confident prediction by training the network with a longer time. However, it is still an efficient way for guiding us on choosing a better network that leads to a high validation accuracy. Table 6 also shows that with more convolutional layers, the model can reach the transition point more quickly. This means that for many deep networks, we can predict the networks’ quality and choose a better model in a short time.

### 3.5. Evaluating DNN’s Capability of Recognizing Objects from Different Classes

The previous sections evaluated neural networks on a whole dataset. However, this evaluation can only test the performance of the network on all the classes. In this section, we will show how to evaluate DNNs on a single class in the information plane when performing image classification tasks.

**Figure 8:**
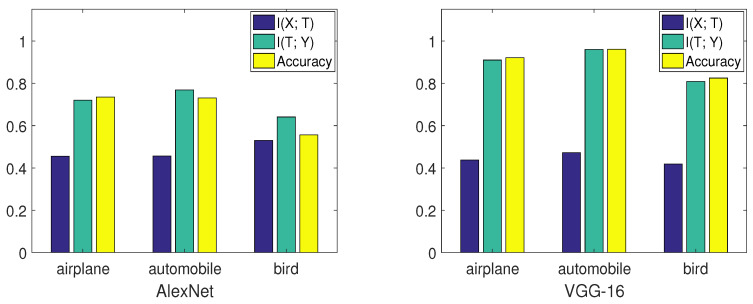
This figure (from [15]) lists I(X;T), I(T;Y) and accuracies for well-trained AlexNet and VGG-16. The accuracy is defined as the percentage of how many samples are correctly predicted out of all samples belonging to that class. For better visualization, we divided I(X;T) by its upper-bound H(X) so that I(X;T), I(T;Y), and the validation accuracy had the same magnitude.

**Figure 9:**
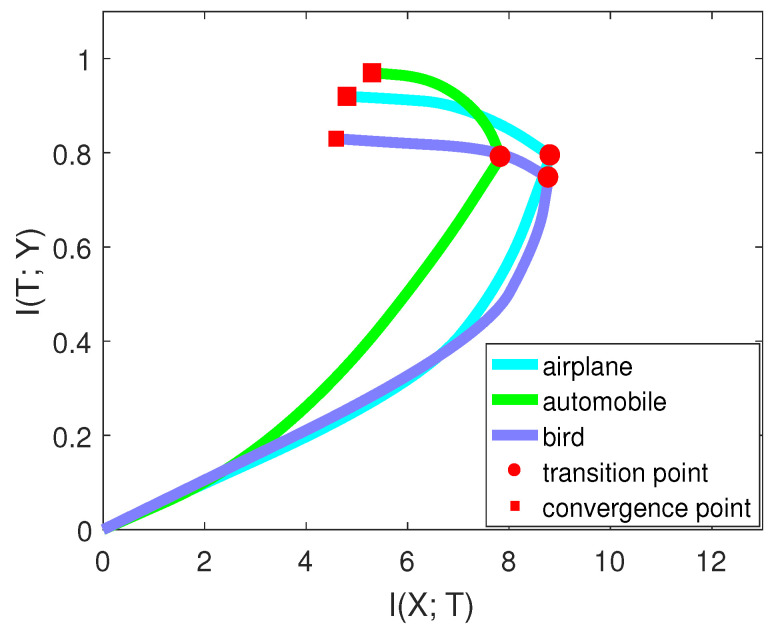
This figure (from [15]) shows the mutual information curves of each class on the CIFAR-10 dataset based on VGG-16.

Suppose there are *C* classes in the dataset and $C_{i}$ denotes the *i*th class. To evaluate networks on the *i*th class, we labeled other classes as one class. Thus, the dimension of label *Y* changes from *C* to two. We also made label *Y* balanced when calculating mutual information so that H(Y) is equal to one. This process is similar to one-vs.-all classifications [31]. However, instead of concentrating on the accuracy, we paid attention to the mutual information. Furthermore, notice that we did not change the network structure; we only performed pre-processing on the validation data. The training scheme and other conditions did not change during this process. We selected three classes (airplane, automobile, bird) on CIFAR-10 to perform the experiment. Figure 8, from [15], compares the performance of two networks on these three classes. Figure 9, from [15], shows the mutual information curve on each class in the information plane.

By comparing the mutual information curves of automobile and airplane, we can find that they had almost the same value of I(T;Y) at the transition point. However, the slope of automobile was smaller than airplane. Thus, the final validation accuracy of automobile may be higher than airplane. By comparing the mutual information curves of airplane and bird, we can find that they almost had the same slope of mutual information curves in the second phase. However, I(T;Y) of airplane was higher than that of bird. Thus, the final validation accuracy of airplane may be higher than bird. The true validation accuracies were 0.921 (airplane), 0.961 (automobile), and 0.825 (bird), which are consistent with our analysis.

Furthermore, we examined two models on the same classes. From Figure 8, VGG-16 had better performance than AlexNet on each class by comparing I(X;T), I(T;Y), and validation accuracies. Thus, the information plane also provided a way to analyze the performance of the network on each class in the image classification task.

### 3.6. Evaluating DNNs for Image Classification with an Unbalanced Data Distribution

In a multi-class classification problem, each class may have different numbers of samples. Suppose we want our model to have a balanced classification capability: we need to control the number of training samples for each class. We suggest that the information plane could help in a quick way.

**Figure 10:**
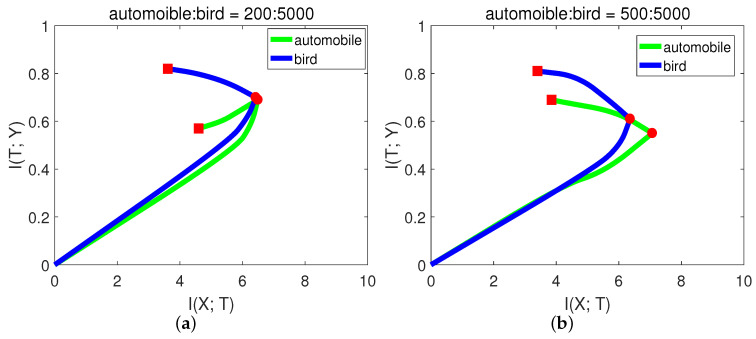

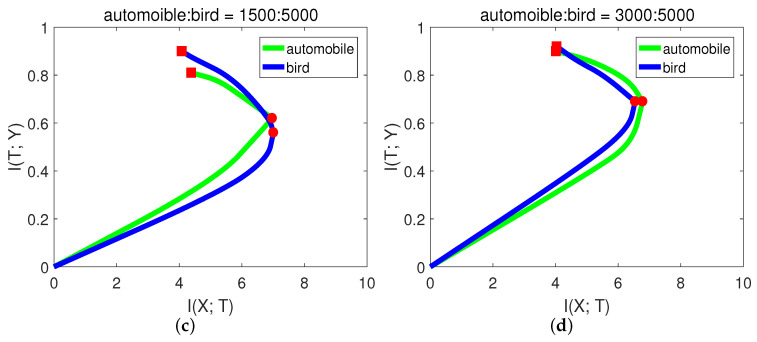
Mutual information paths of automobile and bird with variant numbers of training samples on the CIFAR-10 dataset. The numbers of automobile and bird are (**a**) 200:5000, (**b**) 500:5000, (**c**) 1500:5000, and (**d**) 3000:5000.

In CIFAR-10, each class has 5000 training samples. For simplicity and better presentation, in this experiment, we chose the classes of “automobile” and “bird”. We hoped that the model had balanced classification capability for these two classes. Let the number of training samples for bird be fixed, and we performed the experiments with variant numbers of samples for automobile. The results are shown in Figure 10 and Table 7.

From Figure Figure 10a, when there were only 200 samples of automobile, the I(X;T) of automobile even decreased in the second stage. In this case, the network may just over-fit the small sample noise. From (b) and (c), the slope of automobile in the second phase was higher (negative) than bird, indicating low generalization capability. While when automobile:bird = 3:5, from Figure 10d, the generalization capability of automobile was already comparable to bird. The final validation accuracies from Table 7 of these two classes are consistent with the generalization capability in Figure 10. Since the first phase only took a little time, we could decide how many samples were needed by checking the slope in the second phase.

We believe this application has huge potential in some serious areas, such as medical science, especially where people expect computers to automatically classify diseased tissues from samples. For example, at present, cancer classification has two major problems: the healthy and diseased samples are very unbalanced; the AI system lacks interpretability. Since IB explains the network from an informative way and our framework shows we could determine the number of samples for each class in a short time, our framework could be applied to medical science in the future.

### 3.7. Transfer Learning in the Information Plane

Using the pre-trained model to train data instead of training a neural network from scratch has been common in recent days. It may greatly reduce the training time and make the model easy to learn. In this section, we study why the pre-trained model is better in the language of the information plane.

**Figure 11:**
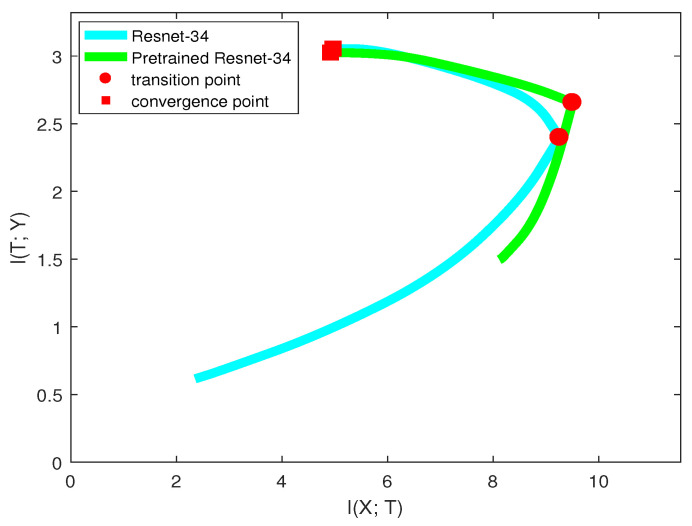
Mutual information paths in ResNet and pre-trained ResNet.

We used the same neural network structure (ResNet-34) as our base model and pre-trained model. The pre-trained model was trained on the ImageNet dataset. We resized the images of CIFAR-10 to 224×224 for fitting the network structure. Figure 11 shows the mutual information paths in the information plane.

We can see for these two models that they had very similar curves in the “compression” phase and ended up with the same convergence point. However, in the “fitting” phase, they acted differently. The pre-trained model had larger I(X;T) and I(T;Y) at the initial time, which contributed to the similarity between CIFAR-10 and the ImageNet dataset. Thus, pre-trained model reached the transition point much faster than the base model. After the transition point, since they had the same architecture, the generalization capabilities for these two models were the same.

Therefore, the information plane revealed that pre-trained model improving learning algorithm happened in the “fitting” phase. and it helped the model fit labels efficiently. However, the pre-trained model and the base model without pre-training may have equal generalization capability. Interestingly, our result is consistent with a recent paper [27], where Kaiming He et al. found that ImageNet pre-training is not necessary. ImageNet can help speed up convergence, but does not necessarily improve accuracy unless the target dataset is too small. Differently, we verified this result by utilizing the information plane.

### 3.8. SGD versus Adam in the Information Plane

Choosing an appropriate optimization method is essential in the neural network training process. A straightforward way to optimize the loss of DNN is via stochastic gradient descent (SGD). However, the loss function of DNN is highly non-convex, which makes SGD easily stuck in the local minima or saddle points. Thus, several advanced optimization methods were developed in recent years and have become popular. Momentum SGD [32] is a method that helps accelerate SGD in the relevant direction and dampens oscillations. The work in [33] adapted the learning rate and performed smaller updates for parameters, which could improve the robustness of SGD. The work in [34] proposed Adam, an optimization method that has been generally used in deep learning. Adam also keeps an exponentially-decaying average of past gradients, similar to momentum. Whereas momentum can be seen as a ball running down a slope, Adam behaves like a heavy ball with friction, which thus prefers flat minima in the error surface.

**Figure 12:**
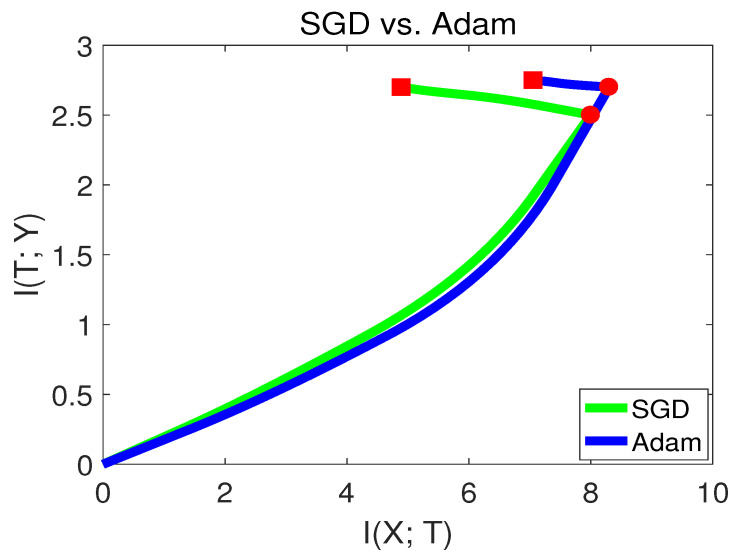
Mutual information paths of SGD and Adam in the information plane.

However, recently, there have been some criticisms about Adam [28]. It was stated that Adam may have better performance at the beginning of the training, but may end up with lower accuracy than SGD. To verify this phenomenon, we visualize SGD and the Adam method in the information plane in Figure 12.

From Figure 12, we can see that Adam had a better capability of fitting the labels in the first stage. while in the second stage, SGD behaved better than Adam. Since SGD converged to a point with lower I(X;T), this indicates that SGD had better generalization capability than Adam at the end of training. Our finding is consistent with [28]. The experiments from Section 3.7 and Section 3.8 show that the information plane can be used as a tool to analyze variant deep learning algorithms.

## 4. Conclusions and Future Work

Our work from the paper aimed to bridge the gap between the theoretical understanding of neural networks and their applications by visualizing mutual information. We summarize our work as follows:

- We investigated the relationship among I(X;T), I(T;Y), and accuracies. The experiment from Section 3.1 showed that low I(X;T) and high I(T;Y) both contributed to the validation accuracy when *X* represented the validation input. Based on this exploration, we proposed a framework by utilizing mutual information to evaluate neural networks. The model with lower I(X;T) and higher I(T;Y) in the second stage of the learning process was more likely to achieve a higher accuracy. The benefit of mutual information is that it provides two terms: I(X;T) and I(T;Y). Compared to the “accuracy”, mutual information can better explore the network’s representation in terms of the capability of fitting the labels and the capability of generalization.
- There were some interesting phenomena when we compared fully-connected neural network (FCs) and convolutional neural network (CNNs) in the information plane. For example, sometimes, the network may not show the second learning stage in the mutual information paths. This happens when a dataset is harder to recognize and the network has fewer layers. It cannot be shown by simply visualizing the loss curve. Thus, we suggest that the information plane is a more powerful tool than loss for evaluating neural networks.
- Since the model with lower I(X;T) and higher I(T;Y) had a big probability of achieving a higher accuracy, $\frac{\Delta I(T;Y)}{\Delta I(X;T)}$ can represent the model’s momentary learning capability in the second stage. The first learning stage only took a little time compared to the whole learning process. We can compare each model’s $\frac{\Delta I(T;Y)}{\Delta I(X;T)}$ at the beginning of the second stage and select a better one. Thus, the information plane could facilitate a quick model selection for a specific problem.
- From Section 3.5 and Section 3.6, with some preprocessing techniques on the data, the information plane can also reveal the recognition capability of each class of neural networks. Thus, we can use the information plane to deal with the classification problem with an unbalanced data distribution. By comparing the mutual information curves of each class, we can determine how many samples are needed to train a classifier with balanced recognition capability.
- There are many sub-fields in deep learning research. We suggest that the information plane is a powerful tool for analyzing particular problems in these sub-fields. For example, weight-transfer learning is often regarded as a technique that improves the model performance; while from our visualization in Section 3.7, weight-transfer learning had almost equal generalization capability compared to the original model without pre-training. This observation is consistent with a recent study in [27]. However, the information plane draws this conclusion in a more informative way. Furthermore, optimization methods are essential in neural networks’ training process. We compared SGD and Adam in the information plane to show how each optimization method behaved during the training in Section 3.8.

There are some future directions based on our current work:

- The mutual information I(X;T) and I(T;Y) is estimated from the last layer of CNNs. One future work is to develop techniques to estimate the information of each kernel in hidden layers with regard to the input and output. This process may help us determine how many hidden layers or how many kernels are needed for designing the network structure. Furthermore, the visualization of hidden units may facilitate a better understanding of neural networks.
- We used the binning technique to estimate the mutual information. This estimation method has limitations, since the bin size and the dimension of the layer may affect the value of mutual information. Some other estimation techniques, like *K*-nearest neighbor [35] and the kernel-density-based estimator [36], are also affected by the dimension of the layer. A possible future work can be done to develop more efficient estimation techniques to stabilize the calculation of mutual information.

## Figures and Tables

**Table 1:** In this table (from [15]), we include 600 samples when performing experiments. The percentage already converged for 600 samples. SGD represents stochastic gradient descent, which means that we stochastically selected a mini-batch from all training samples each time for training the network. BGD represents batch gradient descent, which means we selected all the training samples for training the network. For equal comparison, BGD and SGD used the same training set. CNN-9 is a convolutional neural network with 9 hidden layers. The linear network is a fully-connected neural network whose activation function is the identity mapping.

Network Structure	Training Method	Percentages with 600 Samples
CNN-9	SGD	0.865
	BGD	0.821
Linear Network	SGD	0.755
	BGD	0.594

**Table 2:** This table (from [15]) lists the percentages for convolutional neural networks with variant numbers of hidden layers. CNN-*i* is a deep convolutional neural network with *i* convolutional layers.

Network Structure	CNN-2	CNN-4	CNN-9	CNN-16
percentage with 600 samples	0.56	0.68	0.87	0.96

**Table 3:** This table (from [15]) lists the percentages with the numbers of samples on the validation set. The training scheme and the network structure were SGD and VGG-16, respectively.

Number of Samples	100	200	300	400	500	600
percentage	0.905	0.921	0.912	0.924	0.924	0.924

**Table 4:** This table (from [15]) lists I(T;Y), I(X;T), training epochs, and validation accuracies of each network from Figure 5. The transition points of FC-3, CNN-2, and CNN-4 are just the convergence points, since they did not show the second phase.

Dataset	Model	Transition Point				Convergence Point			
		I(T;Y)	I(X;T)	Epochs	Accuracy	I(T;Y)	I(X;T)	Epochs	Accuracy
MNIST	FC-3	2.96	7.183	1	0.836	3.259	4.358	51	0.983
	FC-6	2.962	7.532	1	0.846	3.249	3.746	56	0.988
	FC-9	2.803	7.166	1	0.774	3.214	3.647	54	0.988
	CNN-2	2.952	7.898	1	0.75	3.282	3.916	50	0.99
	CNN-4	2.286	7.683	1	0.451	3.284	3.621	53	0.994
	CNN-6	2.236	6.184	1	0.515	3.275	3.592	54	0.994
CIFAR-10	FC-3	2.671	10.085	65	0.534	2.671	10.085	65	0.534
	FC-6	2.604	9.321	20	0.537	2.218	7.197	66	0.575
	FC-9	2.55	9.02	21	0.555	2.218	7.197	66	0.56
	CNN-2	1.816	8.133	63	0.451	1.816	8.133	63	0.451
	CNN-4	2.840	8.761	67	0.705	2.840	8.761	67	0.705
	CNN-6	2.301	8.891	5	0.52	2.472	4.862	66	0.781

**Table 5:** This table (from [15]) lists the percentages of each network from Table 2.

Network Structure	CNN-2	CNN-4	CNN-9	CNN-16
percentage of 600 samples	0.56	0.68	0.87	0.96
final accon validation set	0.45	0.70	0.77	0.89

**Table 6:** This table records I(X;T), I(T;Y), training epochs, and model accuracy at the transition point and convergence point for every neural network.

	Transition Point				Convergence Point			
	I(T;Y)	I(X;T)	Epochs	Accuracy	I(T;Y)	I(X;T)	Epochs	Accuracy
AlexNet	2.496	9.476	5	0.524	2.339	5.907	62	0.673
VGG-16	2.262	8.862	7	0.451	2.733	4.682	69	0.839
ResNet-50	2.387	9.389	2	0.541	2.989	4.956	55	0.877
DenseNet-121	2.702	8.965	1	0.604	2.96	4.492	51	0.902

**Table 7:** Final validation accuracies for automobile and bird with different training samples.

Number of Samples (automobile:bird)	200:5000	500:5000	1500:5000	3000:5000
automobile	0.55	0.68	0.85	0.91
bird	0.90	0.91	0.90	0.90

## Appendix A. Experiment Settings

This section introduces the detailed experiment settings in the paper.

(1) In Section 3.1, Section 3.2 and Section 3.3, the networks include FCs and CNNs. The setting for each network is listed in Table A1.
  **Table A1:** Network settings for Section 3.1, Section 3.2 and Section 3.3.
  	Hidden Layers	Batch Size	Initial lr	lr Decay	Optimizer	Momentum
  Linear Network	9	128	0.1	0.1//60	SGD	0.9
  FC-3	3	128	0.1	0.1//60	SGD	0.9
  FC-6	6	128	0.1	0.1//60	SGD	0.9
  FC-9	9	128	0.1	0.1//60	SGD	0.9
  CNN-2	2	128	0.1	0.1//60	SGD	0.9
  CNN-4	4	128	0.1	0.1//60	SGD	0.9
  CNN-6	6	128	0.1	0.1//60	SGD	0.9
  CNN-9	9	128	0.1	0.1//60	SGD	0.9
  CNN-16	16	128	0.1	0.1//60	SGD	0.9
(2) In Section 3.4, we visualize popular networks in the information plane. The network settings are listed in Table A2.
  **Table A2:** Network settings for Section 3.4.
  	Hidden Layers	Batch Size	Initial lr	lr Decay	Optimizer	Momentum
  AlexNet	4	128	0.1	0.1//60	SGD	0.9
  VGG	16	128	0.1	0.1//60	SGD	0.9
  ResNet-50	50	128	0.1	0.1//60	SGD	0.9
  DenseNet	121	128	0.1	0.1//60	SGD	0.9
(3) In Section 3.5 and Section 3.6, we use AlexNet and VGG to evaluate the classification capability of networks for each class in the image classification task. The network settings are listed in Table A3.
  **Table A3:** Network settings for Section 3.5 and Section 3.6.
  	Hidden Layers	Batch Size	Initial lr	lr Decay	Optimizer	Momentum
  AlexNet	4	128	0.05	0.95	SGD	0.9
  VGG	16	128	0.05	0.95	SGD	0.9
(4) In Section 3.7, we use the information plane to analyze the efficiency of transfer learning. The network was ResNet-34, and its settings are listed in Table A4.
  **Table A4:** Network settings for Section 3.7.
  	Hidden Layers	Batch Size	Initial lr	lr Decay	Optimizer	Momentum
  ResNet-34	34	128	0.1	0.1//60	SGD	0.9
(5) In Section 3.8, we use the information plane to analyze different optimization methods in neural networks. The network was CNN-6, and its settings are listed in Table A5.
  **Table A5:** Network settings for Section 3.8.
  	Hidden Layers	Batch Size	Initial lr	lr Decay	Optimizer	Momentum
  CNN-6	6	128	0.1	0.1//60	SGD	0.9

## References

[1] Krizhevsky A., Sutskever I., Hinton G.E. ImageNet classification with deep convolutional neural networks. Proceedings of the Advances in Neural Information Processing Systems.

[2] Seide F., Li G., Yu D. Conversational speech transcription using context-dependent deep neural networks. Proceedings of the Twelfth Annual Conference of the International Speech Communication Association.

[3] Mnih V., Kavukcuoglu K., Silver D., Rusu A.A., Veness J., Bellemare M.G., Graves A., Riedmiller M., Fidjeland A.K., Ostrovski G. (2015). Human-level control through deep reinforcement learning. Nature.

[4] LeCun Y., Bengio Y., Hinton G. (2015). Deep learning. Nature.

[5] Zhang J., Zong C. (2015). Deep Neural Networks in Machine Translation: an Overview. IEEE Intell. Syst..

[6] Silver D., Huang A., Maddison C.J., Guez A., Sifre L., Van Den Driessche G., Schrittwieser J., Antonoglou I., Panneershelvam V., Lanctot M. (2016). Mastering the game of Go with deep neural networks and tree search. Nature.

[7] Simonyan K., Zisserman A. (2014). Very deep convolutional networks for large-scale image recognition. arXiv.

[8] Schroff F., Kalenichenko D., Philbin J. Facenet: A unified embedding for face recognition and clustering. Proceedings of the IEEE Conference on Computer Vision and Pattern Recognition.

[9] He K., Zhang X., Ren S., Sun J. Deep residual learning for image recognition. Proceedings of the IEEE Conference on Computer Vision and Pattern Recognition.

[10] Zhou B., Khosla A., Lapedriza A., Oliva A., Torralba A. (2014). Object detectors emerge in deep scene cnns. arXiv.

[11] Lu Y. (2015). Unsupervised learning on neural network outputs: With application in zero-shot learning. arXiv.

[12] Aubry M., Russell B.C. Understanding deep features with computer-generated imagery. Proceedings of the IEEE International Conference on Computer Vision.

[13] Zeiler M.D., Fergus R. Visualizing and understanding convolutional networks. Proceedings of the European Conference on Computer Vision.

[14] Bau D., Zhou B., Khosla A., Oliva A., Torralba A. Network dissection: Quantifying interpretability of deep visual representations. Proceedings of the IEEE Conference on Computer Vision and Pattern Recognition.

[15] Cheng H., Lian D., Gao S., Geng Y. Evaluating Capability of Deep Neural Networks for Image Classification via Information Plane. Proceedings of the European Conference on Computer Vision (ECCV).

[16] Vasudevan S. (2018). Dynamic learning rate using Mutual Information. arXiv.

[17] Battiti R. (1994). Using mutual information for selecting features in supervised neural net learning. IEEE Trans. Neural Netw..

[18] Hjelm R.D., Fedorov A., Lavoie-Marchildon S., Grewal K., Trischler A., Bengio Y. (2018). Learning deep representations by mutual information estimation and maximization. arXiv.

[19] Tishby N., Pereira F.C., Bialek W. (2000). The information bottleneck method. arXiv.

[20] Chechik G., Globerson A., Tishby N., Weiss Y. (2005). Information bottleneck for Gaussian variables. J. Mach. Learn. Res..

[21] Strouse D., Schwab D.J. (2017). The deterministic information bottleneck. Neural Comput..

[22] Shamir O., Sabato S., Tishby N. (2010). Learning and generalization with the information bottleneck. Theor. Comput. Sci..

[23] Alemi A.A., Fischer I., Dillon J.V., Murphy K. (2016). Deep variational information bottleneck. arXiv.

[24] Thann T., Nguyen J.C. (2018). Layer-wise Learning of Stochastic Neural Networks with Information Bottleneck. arXiv.

[25] Achille A., Soatto S. (2018). Information dropout: Learning optimal representations through noisy computation. IEEE Trans. Pattern Anal. Mach. Intell..

[26] Shwartz-Ziv R., Tishby N. (2017). Opening the Black Box of Deep Neural Networks via Information. arXiv.

[27] He K., Ross G., Dollar P. (2018). Rethinking ImageNet Pre-training. arXiv.

[28] Keskar N.S., Socher R. (2017). Improving Generalization Performance by Switching from Adam to SGD. arXiv.

[29] Raginsky M., Rakhlin A., Tsao M., Wu Y., Xu A. Information-theoretic analysis of stability and bias of learning algorithms. Proceedings of the 2016 IEEE Information Theory Workshop (ITW).

[30] Huang G., Liu Z., Weinberger K.Q., van der Maaten L. (2016). Densely connected convolutional networks. arXiv.

[31] Bishop C.M. (2006). Pattern Recognition and Machine Learning.

[32] Qian N. (1999). On the momentum term in gradient descent learning algorithms. Neural Netw..

[33] Duchi J., Hazan E., Singer Y. (2011). Adaptive subgradient methods for online learning and stochastic optimization. J. Mach. Learn. Res..

[34] Kingma D.P., Ba J. (2014). Adam: A method for stochastic optimization. arXiv.

[35] Gao W., Oh S., Viswanath P. (2018). Demystifying Fixed *k*-Nearest Neighbor Information Estimators. IEEE Trans. Inf. Theory.

[36] Kolchinsky A., Tracey B. (2017). Estimating mixture entropy with pairwise distances. Entropy.

